# Including Vulnerable Populations in the Assessment of Data From Vulnerable Populations

**DOI:** 10.3389/fdata.2019.00019

**Published:** 2019-06-28

**Authors:** Latifa Jackson, Caitlin Kuhlman, Fatimah Jackson, P. Keolu Fox

**Affiliations:** ^1^Department of Pediatrics and Child Health, College of Medicine, Howard University, Washington, DC, United States; ^2^W. Montague Cobb Research Laboratory, College of Arts and Sciences, Howard University, Washington, DC, United States; ^3^Department of Computer Science, Worcester Polytechnic Institute, Worcester, MA, United States; ^4^Department of Biology, College of Arts and Sciences, Howard University, Washington, DC, United States; ^5^Department of Pediatrics, College of Medicine, University of California, San Diego, San Diego, CA, United States

**Keywords:** inclusion, genomics, COMPAS, African Americans, algorithmic fairness, Samoa, criminal justice

## Abstract

Data science has made great strides in harnessing the power of big data to improve human life across a broad spectrum of disciplines. Unfortunately this informational richesse is not equitably spread across human populations. Vulnerable populations remain both under-studied and under-consulted on the use of data derived from their communities. This lack of inclusion of vulnerable populations as data collectors, data analyzers and data beneficiaries significantly restrains the utility of big data applications that contribute to human well-ness. Here we present three case studies: (1) Describing a novel genomic dataset being developed with clinical and ethnographic insights in African Americans, (2) Demonstrating how a tutorial that enables data scientists from vulnerable populations to better understand criminal justice bias using the COMPAS dataset, and (3) investigating how Indigenous genomic diversity contributes to future biomedical interventions. These cases represent some of the outstanding challenges that big data science presents when addressing vulnerable populations as well as the innovative solutions that expanding science participation brings.

## Introduction

The past several decades have seen great improvements in the scale of data collected, analyzed and used to improve human life. This data expands our understanding of social science, business and biomedical science among other disciplines (Murdoch and Detsky, 2013). It is able to find patterns in extensive data sets, and use those observations to test hypotheses and predict phenomena. Unfortunately, the vast majority of this data has been focused on a small subset of global ethnic diversity and culture. In particular, the dominance of European and Asian data science culture has skewed both data science analysis and inference. In the analysis of social science data, the global consequences of social exclusion are costly, including exacerbating poverty, reducing human capital and diminishing culturally coherent solutions which could be more easily adopted in communities (Tangcharoensathien et al., 2018). Meanwhile, a scan of biomedical data shows consistent inequalities in the inclusion of those vulnerable populations that are at most risk for having health disparities (Popejoy and Fullerton, 2016). Indeed the need for better data collection, reporting, analysis and interventions on the environmental and social determinants of health is pressing, and improvement may influence patient health outcomes (Lu et al., 2018).

There is however a critical absence of discussion around the role that vulnerable populations themselves play in articulating the data science problems. This perspective is crucial for designing analytical solutions and most relevantly in interpreting findings from their culturally competent lens. This lack of engagement leads to loss of agency in problem identification, under-representation in the analytical data science space and ultimately poorer solutions that fail to take into account the lived experiences of vulnerable populations.

We seek to use three case studies to explore ways that data scientists, human geneticists, and biological anthropologists can collaborate to encourage the participation of vulnerable populations in data science to address locally relevant questions, generate novel datasets, and learn how to address systemic biases in currently existing datasets. Here we highlight three approaches to involving members of vulnerable populations in the collection, analysis, and interpretation of data derived from vulnerable populations. Each case study explores how vulnerable/ethnic minority populations can be engaged to contextualize data inference within a social context to bring better understanding. In the first example, we introduce work by researchers at Howard University, in remediating the paucity of genetic knowledge about African-descended groups and ameliorating their consequent health vulnerabilities. The second example describes our experience training vulnerable populations about criminal justice data to gain their insights into what that data might mean for their communities. The third example looks at the impact of the exclusion of vulnerable Polynesian populations in variant identification for obesity pharmacogenomics based on biomedical sample collection. Each case study highlights how vulnerable population can make meaningful contributions to the assessment and interpretation of big data. While these case studies do not provide a complete solution to the lack of participation of vulnerable populations in their well-being, they do chart a roadmap that show how engagement can lead to higher quality data generation, new dataset construction, and community trust-building and empowerment in data science.

## Case 1: Generating Genomic Data Equity in Vulnerable Populations

In spite of the origins of humanity in continental Africa and the ancient, historical, and contemporary dispersions of African peoples via at least four major Diasporas worldwide (Zeleza, 2005), very little is known about the genesis, extent, and duration of African genetic variability. This scientific reality increases the vulnerability of modern African-descended populations and limits their ability to benefit from new advances in genomic sciences (Sirugo et al., 2019). The benefit of modern genomics is primarily through the development of comprehensive and inclusive reference databases to which newly discovered variants can be compared and contextualized (Jackson, 2018). Effective genetic medicine depends upon such reference databases. Without appropriate reference standards, the push for subpopulation relevant precision medicine invariably falls short and the targeted population remains under-served and sometimes dis-served. Furthermore, there is an ongoing urgent need to see Africa on its own terms as terrain of the endogenous and the indigenous, a locale of emergence whether its genetics, morphology, ecology, language/linguistics or culture (writ large) (Keita personal communication 2019). This can only be done by integrating scientists and other scholars from the understudied indigenous communities to actively participate in the collection, analysis, interpretation, and dissemination of genetic knowledge about their own people.

Currently, the reference databases are predominantly Eurocentric, as are the genomic priorities in mainstream western science. This is expected and not problematic in and of itself since the majority of researchers are of European descent. However, this imbalance presents issues when the client base is ethnically and geographically diverse and decidedly non-European. These groups can only benefit from the existing databases to the extent that they maintain genomic profiles congruent with North Atlantic European patterns. In other cases, there may also be population-specific mutations in understudied populations that cause health disparities that go under-diagnosed in African-descended groups (Sirugo et al., 2019) primarily because they differ in mutation patterns from the majority European population. Finally, the interpretation of African-derived genomic data suffers if knowledgeable African and African-descent scholars are not involved in the analysis, contextualization, and practical application of the resulting data.

At Howard University, we have launched three African Genome Projects. In the Atlantic African Diaspora Genome Project our aim is to provide historically-informed, geospatially diverse sampling to the study of African-descended peoples in the America hemisphere. The Atlantic African Diaspora Genome Project aims to collect samples from North, South, and Central America and the Caribbean (*N* = 1,000 samples) (Mann, 2001). The second of the African Genome Projects focuses on continental Africa (*N* = 10,000 samples). This project aims to effectively capture the magnitude of genomic variability in the homeland of humanity by focusing on the various terrestrial biomes on the continent and sampling proportionately from each based on the level of existing ecological complexity. The third phase of our data base development efforts is the Red Sea African Diaspora Genome Project (*N* = 1,000). This effort aims to trace the migration pathways of African-descended groups eastward across the Red Sea and Indian Ocean (Harris, 1971, 2003; Cooper, 1977; Alpers, 1997; Ewald, 2000). This database will allow researchers to track relevant African signals to the east of Africa, following the many well-established historical routes out of the continent. The W. Montague Cobb Research Laboratory has been in the forefront of the development of augmented genomic data bases to characterize African genomic diversity. Our hope is that by acquiring and interpreting representative African genomic diversity, we will develop the capacity to reconstruct the evolutionary history of African descended peoples worldwide and that of our species, and in so doing, increase the access of African-descended populations to the immediate and long term benefits of genomic knowledge.

As the largest and most well-known historically Black university, Howard University is uniquely poised to initiate this study. In this preliminary collection effort, several weeks were devoted to community education and recruitment. We assembled a team of primarily African-descended interdisciplinary researchers to design and implement the project. These scholars included colleagues in the life sciences, medical sciences, social sciences, humanities, and computational sciences. On the day of collection, within 8 h, 463 non-hospitalized individuals freely provided informed consent for access to their DNA, salivary microbiome, ancestral background, and general health information. African Americans from North America and the Caribbean and continental Africans were the pre-identified target populations. While a total of 25 nationalities and 35 ethnicities were represented in this first sample, 260 of our participants (56.2%) self-reported as North American Black or African American. Participant data were subdivided based upon ancestral origins. Three hundred forty-eight participants (75.2%) contribute to the Atlantic African Diaspora Genomes Database, 31 participants (6.7%) from continental Africa will be included in the Continental African Database, and 75 participants (16.2%) will go into the Red Sea African Diaspora Database. Nine participants (2.0%) identified their ancestral origins in Eurasia or Oceania and were assigned to a Control cohort.

The vulnerability of African-descended populations to missing insights and benefits of advances in genomic sciences is particularly acute for continental Africans. These populations retain high levels of regionally specific genetic diversity. Yet, the efforts to date have generally been based on opportunistic sampling of Africans. Consequently, for more continental Africans, current genomic knowledge is particularly non-illuminating. Without carefully constructed reference genomic databases that integrate ecological, anthropological, and historical data what is currently known presents a weak profile of continental African substructure, population stratification, and migration history. The ability to reconstruct the biological histories of Africans remains limited and with only a few selected African populations studied, our knowledge of continental African diversity lacks the nuanced regional and ethnic specificity that characterizes European reference databases. If African genetic diversity was studied systematically, we expect it to yield as much, if not more, geospatial and ethnic complexity as Europe. In particular, since humans have had a protracted residence in Africa, there have been ample opportunities for regional adaptations to emerge, and extensive migrations throughout the continent have occurred over hundreds of thousands of years.

Very limited genomic studies of indigenous Africans have been done and even fewer are publicly available and integrated in general reference databases for comparative research purposes. Although the 1000 Genomes Project (1000GenomesConsortium, 2015) reconstructed the genomes of 2,504 individuals from 26 populations using a combination of low-coverage whole-genome sequencing, deep exome sequencing, and dense microarray genotyping, Africa was not adequately represented given its status as the homeland of our species, continent of longest residence, and therefore the indigenous peoples with the greatest expected collective accumulations of acquired mutations. Although the 1000 Genomes Project characterized a broad spectrum of genetic variation, in total over 88 million variants [84.7 million single nucleotide polymorphisms (SNPs), 3.6 million short insertions/deletions (indels), and 60,000 structural variants], all phased onto high-quality haplotypes, coverage of the non-European populations from whom many American lineages can be traced remains insufficient, particularly given the long presence of African-descended individuals in this hemisphere, the extensive opportunities for gene flow with non-Africans, and the continentally diverse origins of these early Africans to America. This was noted over 20 years ago Jackson (1996, 1997, 1998), yet the deficiency in our databases persists.

For Diaspora African populations such as Legacy African Americans who have been in the country for 11–16 generations and are an amalgamation of African peoples with modest gene flow from non-Africans, more information is known about the European-derived components of their genomes than is revealed about their larger, residual African components. This limits the value of current genomic medicine in these individuals. Furthermore, since much of this admixture with Europeans occurred within the context of African enslavement in the seventeenth, eighteenth, and nineteenth centuries, the European-derived segments in the genomes of African Americans tend to be truncated in length and random in their dispersion in the genome. Although an estimated 30% of Legacy African American men carry Y-chromosome haplogroups found more commonly in North Atlantic Europe, the rest of their genomes also reflect this historical European admixture, but the distribution of these genes is non-uniform and piecemeal.

The historically most important diaspora for African people has been inadequately studied. This is the intra-African diaspora. Unfortunately, however, knowledge of the genomic and demographic ramifications of intra-African migrations, adaptations, and admixtures are lacking. For the vast majority of continental Africans and African descended people outside of Africa, the more African their lineage, the less current genomic knowledge is able to reveal about their disease vulnerabilities, ancestry, and phenotypic markers. The ramifications of inadequate studies of African genomic diversity are not limited to individuals of African descent. In previous studies we have shown that personalized genomic testing can have multiple beneficial educational ramifications for tested individuals (Johnson and Jackson, 2015). Even a small amount of data on one African ancestry has been shown to stimulate additional interest in this history and the science behind it. In the absence of relevant information, these opportunities, for example in enhanced interest in STEM, are diminished.

Our approach can remediate this situation and bring equity to our genomic knowledge by capturing a wider diversity of human variability. A first step has been to increase the number of diverse non-European individuals in the reference databases, creating truly comprehensive and representative databases for meaningful world-wide comparisons and as a platform for broadly beneficial precision medicine. A particular need is to capture the high variability of indigenous Africans in each of the terrestrial biomes of the continent, since much of this genomic diversity is not yet characterized. This has to be done in an intentional model-based sampling method, not haphazardly or simply opportunistically. Sampling should also not be biased toward hunter-gatherer groups to the exclusion of agriculturalists and post-agriculturalists in Africa. We need clear hypothesis-driven sampling strategies for studying genomic diversity in non-European peoples and these need to be coupled with relevant historical, anthropological, ecological, and geospatial data. These data should be integrated using computational biology to generate algorithms that accurately characterize the populations under study, reconstruct their histories, and provide predictive data for their enhanced survival.

To generate sophisticated bioinformatic profiles of African genomic diversity, we need to identify the salient population substructure of African and African-descended donors so that their genomics can be appropriately contextualized. Using ethnogenetic layering in the Atlantic African Diaspora, we have hypothesized that microethnic groups such as the Gullah/Geechee of the South Carolina Lowcountry may retain unique genomic markers as a consequence of their antiquity (compared to other African American groups), relative geographic and cultural isolation (Jackson, 2008), and endogamous mating preferences (Caldwell, personal communication). This is not only due to the geographical distances between these groups, but also because of their differing population histories, migration stories, admixture patterns, dietary exposures, and other relevant variables.

In collaboration with Helix and National Geographic, our strategy is to divide the completed data bases equally into Discovery and Replication cohorts for the integrative testing of hypotheses regarding admixture, ancestry, migration, selection, disease susceptibility/resistance. Once completed, these databases will provide the scientific community with greater referencing depth with expected positive ramifications for a public increasingly interested in and dependent upon the results of genomic interpretations for their health and well-being. This case emphasizes the need to form substantive collaborations with institutions such as Howard University that are addressing questions related to the health of underrepresented populations. We are interested in forming collaborative relationships with data scientists to develop appropriate analytical algorithms for population inference.

## Case 2: Encouraging Participation of Vulnerable Groups in Data Science for Algorithmic Fairness

In this case study, we describe our experience working with criminal justice recidivism data to design a tutorial for the Broadening Participation in Data Mining workshop (BPDM). The tutorial on algorithmic fairness in the criminal justice system took place at BPDM 2019, a 3-day standalone workshop for 65 underrepresented gender, ethnicity and ability minorities from undergraduate through early career data scientists held at Howard University. This algorithmic fairness tutorial was first introduced by Dr. Falvio Calmon from Harvard University at BPDM2017 in Halifax, Nova Scotia. The 2017 co-location of BPDM with SIGKDD and the Fairness Workshop increased BPDM participant exposure to the topics of algorithmic fairness and data mining. Each tutorial has been preceded by a panel discussion on algorithmic fairness and the role of data scientist derived from vulnerable populations in recognizing the underlying biases inherent in large data sets such as the COMPAS dataset. The tutorial introduces the topic of algorithmic fairness, which attempts to identify and mitigate unfair bias against vulnerable groups in automated decision making procedures, and investigates in-depth the application of one such automated tool within the criminal justice system in the US.

We feel there are a number of benefits to focusing the hands-on tutorial for the BPDM workshop on this topic. Teaching tools that incorporate social good topics (in this case social justice, criminal justice, and algorithmic fairness) have been identified as having potential for broadening participation in computing (Buckley et al., 2008) where women and ethnic minorities have been woefully underrepresented. Students motivated by their interests and values, and engagement with non-traditional students can tap into this by demonstrating ways that computer science can have a positive social impact and “make a difference” (Goldweber et al., 2013). In addition to appealing to their interests, exposing students to the topic of algorithmic fairness can advance their research skills, exposing them to cutting-edge research practices for real world competency, and ethical application of data mining skills.

Furthermore, creating a more inclusive body of data analysts looking into this type of problem data can help ensure a diverse and inclusive critical perspective on the use of AI in society. Participants at the BPDM workshop are members of underrepresented groups in computing, representing ethnic, ability and gender minorities identified as vulnerable populations on both side of the data analysis pipeline. A key consideration of our tutorial development was to avoid putting the burden of addressing unfair structural biases onto the very members of the populations who are being made vulnerable. However, given the recent research interest in judicial fairness, our workshop provided the opportunity for trainees from vulnerable populations to use data as a means to both identify structural inequalities and to address those inequalities using algorithmic fairness nested within a social equity construct.

Interest in the impact of big data on society has been growing recently in the data mining and machine learning community, with input from legal scholars (Barocas and Selbst, 2016). Of particular concern is the use of algorithmic decision making procedures in regulated domains such as lending, housing and criminal justice. The study of algorithmic fairness seeks to address the fact that structural inequities which exist in our society can be encoded in subtle ways in the data we collect and analyze, allowing discriminatory practices to be perpetuated or even exacerbated by predictive models trained on historic data. Recent research in the AI community has focused on identifying bias against protected groups, as defined by sensitive data attributes such as age, gender, disability and ethnicity. Many tests have been proposed for assessing fair outcomes (Hardt et al., 2016; Chouldechova, 2017; Kleinberg et al., 2017). Identifying unfair treatment of these vulnerable populations is a paramount and challenging task, given the widespread use of sophisticated, difficult to interpret, and often proprietary models for decision making.

Recent reporting has, in part, been fueled by a high profile expose (Angwin et al., 2016) published by ProPublica, a Pulitzer prize-winning investigative journalism organization. The article investigates risk assessment tools widely used in the US criminal justice system. These tools are algorithms developed by private companies and purchased by states to evaluate defendants. Judges are presented with risk assessment scores rating the dangerousness of defendants, which they can use in decisions such as setting bail or deciding sentencing. The authors show that one popular tool, called COMPAS, assessed African Americans and European Americans differently when assigning risk scores to be used at bail hearings. Their analysis showed that “black defendants were nearly twice as likely to be misclassified as higher risk compared to their white counterparts.” In response, the company which developed the algorithm published a counter analysis, using a different statistical test to demonstrate fairness with respect to ethnic inference of a vulnerable population. Computer science researchers then picked up the investigation, publishing numerous results, including showing that the different standards used to determine fairness were impossible to satisfy concurrently Chouldechova (2017); Kleinberg et al. (2017).

The data released with this article has become the de facto benchmark for “fair” algorithms seeking to ensure equal treatment of different groups. The COMPAS dataset is unusual in that it contains real world data demonstrating a direct impact of algorithmic decision making on individuals. The data were available as part of public records, and include sensitive data attributes of race, gender, and age, as well as identifying information. Its popularity and availability have meant it has been used extensively by researchers in a very short time. Choosing this dataset for a hands-on tutorial session at the broadening participation workshop created an opportunity for discussion and reflection on the role of members of vulnerable populations as both data points and as data scientists. The tutorial presented a brief overview of the topic, introducing the concepts of protected groups defined by sensitive data attributes such as race and gender. In our workshop discussion we considered the problematic nature of such datasets and their increased role in decision making in our society, alongside other examples. We discuss subtle ways that data have historically been used to enforce discriminatory practices, for example in the redlining practices in which zip code was used as a proxy for race to enforce residential segregation in housing. Then we discuss ways that unfair bias can enter a modern data mining pipeline.

Typical data mining models train on data collected in the past, and then are used to make decisions about the future. If there are historical inequalities inherent in the training data, they will be perpetuated, and possibly even exacerbated by our predictive model. Skewed training data can lead to better accuracy for some groups vs. others. We discussed the example of gender stereotypes encoded in word embeddings used in natural language processing (Bolukbasi et al., 2016), and the example of facial recognition tools trained on majority white, male faces (Buolamwini and Gebru, 2018). These examples demonstrate cases where fairness research had a real world impact, as these papers have prompted companies to improve facial recognition software, and the development of bias mitigation techniques for text analysis. We discussed questions to consider when developing/applying new method, e.g., “Who will use this technology, and will it work equally well for everyone?” and “Is my dataset representative of all groups?”

The learning objectives of the tutorial are to examine some examples of structural inequality in society that is buttressed by data mining practices including developing ways to recognize ways in which unfair bias might be introduced into a data mining pipeline. Because vulnerable populations are often placed in the position of being whistleblowers for structural inequalities, we discussed how to perform analyses to verify whether a predictive model is fair or unfair and what outcomes should be considered when developing data mining techniques beyond accuracy. To address these concerns we have to develop tools to democratize the development of data mining techniques and technologies using open and transparent methods with clearly reproducible findings. This tutorial demonstrates one approach to doing this [i.e., with interactive Jupyter notebooks (Kluyver et al., 2016)] and give students hands-on experience with open software tools.

The Algorithmic Fairness for Vulnerable Populations tutorial steps through a typical data analysis pipeline. First the data is cleaned and preprocessed according to the steps taken in the ProPublica analysis. Then a number of statistical and visualization methods are applied to allow participants to assess the attributes in the training dataset and understand whether there is any unfair bias present. Finally, three notions of group fairness are introduced, covering state-of the-art bias detection metrics from the recent literature:

- Disparate Impact. This legal concept is used to describe situations when an entity such as an employer inadvertently discriminates against a certain protected group. This is distinct from disparate treatment where discrimination is intentional. To demonstrate cases of disparate impact, the Equal Opportunity Commission (EEOC) proposed “rule of thumb” is known as the 80% rule.
- Calibration. This statistical test was used to verify the fairness of the COMPAS model by the company Northpoint that created the tool. The basic idea behind calibrating a classifier is to have the confidence of the predictor reflect the true outcomes. In a well-calibrated classifier, if 100 people are assigned 90% confidence of being in the positive class, then in reality, 90 of them should actually have had a positive label. To use calibration as a fairness metric we compare the calibration of the classifier for each group.
- Equalized Odds. The last fairness metric we consider is based on the difference in error rates between groups. The equalized odds criterion (Hardt et al., 2016) proposes to look at the difference in the true positive and false positive rates for each group. This aligns with the analysis performed by ProPublica.

The goal of this tutorial's implementation was to allow for hands-on analysis right away, without requiring any heavy overhead from installing many tools or having to clean and pre-process the data. At the same time, all analysis was fully transparent and available for experimentation. Participants could step through the notebook and simply follow along, or dig deeper and edit the code directly to experiment with the data. Suggestions for possible further experimentation are provided throughout. Links to datasets, research papers, Wikipedia entries, and Python data mining tools provide context and avenues for deeper investigation into the topics and methods described. A clear outcomes was that trainees who undertook the data manipulation and assessment felt empowered to identify the limitations of data resulting from structural inequalities and to identify mechanisms to address those biases in data.

## Case 3: Investigating Vulnerable Population Specific Variation Using Genome Editing Tools

Indigenous communities represent a classic example of a vulnerable population for whom territorial rights, educational attainment and health status are all under stress. Nevertheless, they remain a subject of keen genomic interest to western scientists. Unfortunately, these largely one-sided cross cultural scientific interactions between Indigenous populations and European ancestried scientists have long been steeped in misunderstanding and mistrust. Cases like the Havasupai Nation's inclusion in stigmatizing mental health research against their will have helped to drive many Indigenous peoples to reassess their willingness to work with non-Indigenous scientists (Garrison, 2013). The development of novel large scale data generation tools have emphasized the voluntary exclusion of Indigenous populations and the paucity of data upon which to gain meaningful insights on Indigenous communities' health and well-being.

The utility of data analysis has been readily adopted by human geneticists, who have willingly accepted the tools of big data to better understand the features of the genome including variable sites across the genome, chromosomal arrangements, and population level variation.

This pursuit of ever increasing data has lead to breakthroughs in ancestry assessments, multi-omic precision medicine models and has spurred molecular breakthroughs like the Crispr-Cas9 system of gene editing. Crispr-Cas9, most recently made infamous by the ethically condemned modification of Chinese twins (Schmitz, 2019).

While genome sequencing is a great tool for identifying genetic variation that might be involved in disease mechanisms, correlation does not equal causality. Gene editing tools offer the population geneticists the opportunity to identify population-specific variation derived from large scale sequencing experiments and to conduct further assessment of the functional significance of genome sequence variation, thus potentially identifying the changeable sites underlying traits or disorders. For example, gene editing technologies can be used to investigate population-specific, positively selected point mutations implicated in a range of diseases (Komor et al., 2016). In addition to using these tools that are already in existence to functionally investigate individual variants in clonal cell lines, multiple laboratories have begun to develop new editing tools to simultaneously introduce multiple mutations in the human genome via multiplex nucleotide editing of population specific haplotypes under selection, or multiple point mutations on different chromosomes in human genome.

Engineering new tools to functionally investigate single nucleotide changes is an exciting prospect for two primary reasons: (A) Creating accountability. Culturally competent empirical evidence and detailed theoretical considerations should be used for evolutionary explanations of phenotypic variation observed in humans (especially Indigenous populations). Population genetics investigators frequently overlook the importance of these ethnographic criteria when associating observed trait variation with evolutionary analysis. Functional investigation of population specific variation has the potential to empower the population genetics community by holding evolutionary explanations accountable (Gould and Lewontin, 1979). This need for mechanistic insight is framed by problematic narratives and exacerbated by correlation based studies that fail to properly functionally investigate single nucleotide changes. Because Indigenous populations are vulnerable (i.e., at risk populations), it is the genomic technology development community's responsibility to take these potentially problematic narratives to task (Neel, 1962). Not to just reclaim Indigenous history through the population genetics projects we champion, but potentially empower Indigenous history with genome editing tools. (B) Democratizing tools. Indigenous peoples are under-represented in both population-based genomic studies, and as primary investigators in academia. For Indigenous researchers, this leads to questions as to how Indigenous peoples will meaningfully participate in human population genetics, and how to address the disparities currently existing in Indigenous communities? One way that Indigenous scientists are addressing this is the formation of an educational consortium that is focused on educating Indigenous genetics, such as the Summer Internship for Indigenous Peoples in Genomics (SING Consortium). This research consortium works with Indigenous communities to generate large scale data to address the genomic and health disparity questions that those communities have Claw et al. (2018).

In addition to standard metrics of academic success such as grant awards and paper publications, Indigenous researchers must transition our research focus to understanding how independent research programs will become actionable. If participating Indigenous communities are not presented with tangible benefits to collaborating with non-Indigenous scientists, such as access to medicine, developments to infrastructure, or capacity building, then research focusing on Indigenous communities could potentially continue a legacy of colonial exploitation. Technological independence, self-governance, and democratization of the tools should always be the long-term goal of ethical partnerships in genomic sample collection, large scale data analysis and inference generation. Some easy solutions to address these concerns include engaging Indigenous communities in educational seminars within Indigenous spaces including Native American Reservations, Hawaiian Heiau, and Maori Marae. Another priority must be to transition genomic research toward focusing on the development of biomedical tools to make gene editing of deleterious genomic changes more affordable, empowering Indigenous populations across the globe to gain agency over their own future.

## Discussion

Each of these case studies demonstrates how vulnerable ethnic and justice status individuals can be involved, not just as the objects of proposed studies of vulnerable populations but in the study design, implementation, and importantly the analysis and inferential assessment of results. In each case, including vulnerable populations can yields better more inclusive results with populations becoming invested in the outcomes of evidence-based analysis. Among the lessons derived from these cases are that partnering with institutions that serve vulnerable populations is crucial to the collection of bias free data. This collaboration must include clear benefit for vulnerable communities. Another lesson learned is that where data on vulnerable populations exists, partnering with data scientists derived from those vulnerable populations can help to disentangle an algorithm's inferential ability from a manifesting of implicit bias in data collection. Finally overcoming generational reluctance to participate in research on underserved populations requires both educational trust building and dialogue with a collaborative spirit. Data science must include vulnerable populations in the research design, analysis and inference of data findings in order to make interpretations that are valuable and meaningful to those populations. Whether focused on social science, biomedical applications or preventing the harvesting of large scale genomic data from vulnerable populations with no clear reciprocal benefit to them, the inclusion of these diverse population and perspectives can improve data science. In addition, the continuing need for broad educational access and enhanced ability to make sense of the increasing complexity of big data requires that more vulnerable community perspectives be included.

While we focus on the role that vulnerable populations can play in addressing the information, health and social justice disparities in their communities, it is equally important to identify the role that intersectionality plays in the lived identities of vulnerable populations. We believe that this is an area that needs to be further addressed in the data science research literature. Taken together, these case studies present illustrative examples of how vulnerable populations, researchers, and the institutions that serve them can contribute to improving data science by their participation, not just as study subjects, but as robust intellectual research partners.

## Data Availability

The COMPAS tutorial datasets analyzed for this manuscript can be found in the GitHub repository https://github.com/caitlinkuhlman/bpdmtutorial.

## Ethics Statement

The African American Genome Projects was carried out in accordance with the recommendations of the Human subjects guidelines of the Howard University Institutional Review Board with written informed consent from all subjects. All subjects gave written informed consent in accordance with the Declaration of Helsinki. The protocol was approved by the Howard University Institutional Review Board.

## Author Contributions

LJ conceptualized the collaborative manuscript theme. LJ, PF, FJ, and CK each wrote sections of this manuscript. LJ and CK edited the manuscript. All authors contributed to manuscript revision, read, and approved the submitted version.

### Conflict of Interest Statement

The authors declare that the research was conducted in the absence of any commercial or financial relationships that could be construed as a potential conflict of interest.
